# Identification of KIF4A and its effect on the progression of lung adenocarcinoma based on the bioinformatics analysis

**DOI:** 10.1042/BSR20203973

**Published:** 2021-01-22

**Authors:** Yexun Song, Wenfang Tang, Hui Li

**Affiliations:** 1 Department of Otolaryngology-Head Neck Surgery, Xiangya Hospital, Central South University, Changsha 410008, Hunan Province, China; 2 Department of Respiratory Medicine, The First Hospital of Changsha, Changsha 410000, Hunan Province, China

**Keywords:** Bioinformatical analysis, Biomarker, Differentially expressed genes, KIF4A, Lung adenocarcinoma

## Abstract

**Background:** Lung adenocarcinoma (LUAD) is the most frequent histological type of lung cancer, and its incidence has displayed an upward trend in recent years. Nevertheless, little is known regarding effective biomarkers for LUAD.

**Methods:** The robust rank aggregation method was used to mine differentially expressed genes (DEGs) from the gene expression omnibus (GEO) datasets. The Search Tool for the Retrieval of Interacting Genes (STRING) database was used to extract hub genes from the protein–protein interaction (PPI) network. The expression of the hub genes was validated using expression profiles from TCGA and Oncomine databases and was verified by real-time quantitative PCR (qRT-PCR). The module and survival analyses of the hub genes were determined using Cytoscape and Kaplan–Meier curves. The function of KIF4A as a hub gene was investigated in LUAD cell lines.

**Results:** The PPI analysis identified seven DEGs including BIRC5, DLGAP5, CENPF, KIF4A, TOP2A, AURKA, and CCNA2, which were significantly upregulated in Oncomine and TCGA LUAD datasets, and were verified by qRT-PCR in our clinical samples. We determined the overall and disease-free survival analysis of the seven hub genes using GEPIA. We further found that CENPF, DLGAP5, and KIF4A expressions were positively correlated with clinical stage. In LUAD cell lines, proliferation and migration were inhibited and apoptosis was promoted by knocking down KIF4A expression.

**Conclusion:** We have identified new DEGs and functional pathways involved in LUAD. KIF4A, as a hub gene, promoted the progression of LUAD and might represent a potential therapeutic target for molecular cancer therapy.

## Background

Lung cancer is one of the most frequent malignant tumors in clinical practice [1]. Its morbidity and mortality have ranked first among all types of malignant tumors, and it has been recognized as the most widespread cancer in the world [2]. More than 80% of lung cancers are non-small cell lung cancers (NSCLC), and lung adenocarcinoma (LUAD) accounts for over 70% of NSCLC [3,4]. In recent years, the treatment of LUAD has continuously improved, and a series of new diagnosis and treatment technologies have been applied in clinical practice, which can improve the treatment outcomes of LUAD patients to some extent [5–7]. Nevertheless, the 5-year survival rate is still very low, and outcomes are still unsatisfactory [8]. At present, early diagnosis and treatment can greatly improve prognosis and survival rates of LUAD patients [9,10]. Therefore, it is crucial to fully elucidate the molecular mechanism involved in LUAD.

In recent years, with the development of microarrays and high-throughput sequencing technologies allowing large open data resources, such as The Cancer Genome Atlas (TCGA) database and Gene Expression Omnibus (GEO) datasets, large amounts of genetic data have been generated [10–12]. Researchers can effectively mine big data to identify novel cancer-related genes [13–15]. At present, how to extract valuable information from this huge database has become a new research direction, and bioinformatics has effectively solved this problem. Bioinformatics can screen for availably and mine the microarray data, and ultimately applies relevant software to graph the results, so as to reveal genes potentially implicated in oncogenesis at the molecular level [16,17]. Consequently, bioinformatics can provide new diagnostic markers and novel ideas for the early diagnosis and treatment of LUAD.

The purpose of the present study was to identify the potential key genes and pathways associated with the carcinogenesis of LUAD. We first compared the gene expression profiles of LUAD and adjacent normal lung tissues utilizing the data in the GEO public database. Next, we systematically analyzed the expression profiles of microarray data and screened DEGs. Meanwhile, we determined the Gene ontology (GO), Kyoto Encyclopedia of Genes and Genomes (KEGG), and protein–protein interactions (PPIs) network analyses of the robust DEGs in LUAD. Further, we also obtained and analyzed the expression and prognosis of the related hub genes using the Oncomine database and the TCGA and GTEx datasets in GEPIA. We validated the mRNA expression of the seven hub genes in clinical samples of LUAD and in paired normal tissues. Furthermore, we investigated the potential functional mechanism of KIF4A in regulating LUAD cell proliferation, migration, and apoptosis.

## Materials and methods

### Data acquisition and preprocessing

Processed microarray data were searched from the websites of GEO (available: http://www.ncbi.nlm.nih.gov/geo/) using “LUAD” as the keyword. Of these, we selected and downloaded the GSE85716, GSE32863, and GSE116959 datasets (Supplementary Table S1). DEGs were identified by the Bioconductor limma package and the robust rank aggregation (RRA) method was used to integrate and rank all of the DEGs from three GEO datasets. In addition, the edgeR package was used to screen DEGs with thresholds of |log2fold change(FC)|>1 and the thresholds of the adjusted p-value false discovery rate (FDR)<0.05.

### Hierarchical clustering analysis

The top 20 robust DEGs were determined using the robust RRA method. As in a previous report [18], the R language package was applied for hierarchical clustering analysis based on the TPM values of the genes in each group. In the analysis, TPM values represented the expression levels of genes, and the gene expression patterns were further clustered into the same or similar genes. In the clustergram, differences in subregional information were represented using different colors, and the different experimental conditions were determined using the clustering pattern control model.

### GO analysis

Direct annotation of gene function can obtain multiple and complicated functional nodes, which can result in the redundancy of results. Thus, we applied the GO analysis method to extract and integrate data and further increase the reliability of the gene analysis. GO analysis mainly includes cellular components (CC), molecular functions (MF), and biological processes (BP). In the present study, GO enrichment analysis of DEGs was also analyzed using the Bioinformatics Tool (DAVID, version 6.8, https://david.ncifcrf.gov/) [19,20].

### KEGG analysis

KEGG analysis, created by the Kanehisa Laboratories, is a collection of databases that can integrate genomes, biological pathways, diseases, drugs, and chemicals [21]. KOBAS 2.0 software (http://kobas.cbi.pku.edu.cn/) was used to carry out KEGG pathway enrichment analysis for the DEGs in LUAD [22]. A *P*-value <0.05 was used as the screening threshold to explore the biological signaling pathways involved in DEGs in LUAD.

### PPI analysis

The STRING (http://string-db.org/) is a database that can search for and predict the interactions between known proteins [23]. The online software string (comprehensive score>0.9) was adopted to analyze the PPIs of DEGs, and then a PPI diagram including the DEGs was drawn based on the analysis. Meanwhile, a number of nodes in the network was obtained to denote genetic relationships through network structure analysis, and the core genes were defined by the number of nodes in the network. Cytoscape software was used to generate the PPI network diagram. The software includes two algorithms: MCC and DMNC, which can calculate the top 10 genes and then cross-cut their intersections. The hub genes were obtained from the PPI network and were then subjected to verification of their expression, survival analysis, and other analyses.

### Application of oncomine database

The Oncomine database (http://www.oncomine.org) was utilized to obtain the expression of seven hub genes in LUAD and in adjacent normal lung tissues. The screening conditions were as follows: (1) Genes: BIRC5, DLGAP5, CENPF, KIF4A, TOP2A, AURKA, or CCNA2; (2) Analysis Type: LUAD versus Normal Analysis; (3) Threshold using *P*-value<0.05; FC: All; Gene Rank: All.

### Gene expression profiling interactive analysis (GEPIA) database

GEPIA is a public database established for expression profiling analysis of cancer and normal genes [24]. GEPIA analysis contains the expression analysis of RNA sequencing data from 9736 tumors and 8587 normal samples in TCGA (http://cancergenome.nih.gov/) [25] and Genotype-Tissue Expression (GTEx, http://commonfund.nih.gov/GTEx/) projects [26,27]. In our study, the expression of the seven hub genes was extracted from the TCGA database. In addition, the survival and disease-free survival analyses of the seven hub genes were also obtained based on TCGA and the GTEx data in GEPIA. Moreover, we also obtained the correlation of CENPF, DLGAP5, KIF4A, and the clinical stage of LUAD.

### Total RNA isolation and real-time quantitative PCR (qRT-PCR)

In total, samples from 10 LUAD patients (along with paired normal lung samples) that had undergone surgical treatment in our hospital, were available for examination in our study. All collected tissues were confirmed histologically by two independent pathologists. Written consent was obtained from all patients who were recruited. This research was approved by the Ethics Committee of the First Hospital of Changsha.

Total RNA from LUAD samples (*n*=10) and paired normal samples (*n*=10) was isolated using the RNeasy Mini Kit (Cat.74101, Qiagen, Germany) according to the manufacturer's instructions. The synthesis of cDNA used for genes was performed using the Bestar™ qPCR RT kit (DBI; #DBI-0) using 2 μg RNA. The relative mRNA levels of BIRC5, DLGAP5, CENPF, KIF4A, TOP2A, AURKA, and CCNA2 were determined by real-time quantitative PCR (qRT-PCR) using a 20 μl reaction system. The PCR process was performed on an ABI PRISM 7500 real-time PCR system (Applied Biosystems, Carlsbad, CA, U.S.A.) using the following settings: 95°C for 2 min, followed by 40 cycle of 94°C for 20 s, 58°C for 20 s, and 72°C for 20s. GAPDH was used as the internal normalized reference gene. The fold change was determined as: 2−ΔΔCt (ΔΔCt = (ΔCt of genes of interest) − (ΔCt of GAPDH). The primer sequences are listed in Supplementary Table S2.

### Cell culture and reagents

The human LUAD cell lines HCC827 and A549 were purchased from AbZyme Biotechnology Inc (Jiangsu, China). The human bronchial epithelial cells Beas2B were purchased from Zhong Qiao Xin Zhou Biotechnology Inc (Shanghai, China). Beas2B cells were grown in DMEM (Invitrogen Life Technologies, Carlsbad, CA, U.S.A.) supplemented with 5% fetal bovine serum (FBS, Gibco BRL, Gaithersburg, MD, U.S.A.). HCC827 and A549 cells were cultured in RPMI-1640 (Hyclone, Logan, UT, U.S.A.) supplemented with 10% FBS. Rabbit monoclonal antibodies against KIF4A were obtained from Abcam (#ab124903, Abcam, Cambridge, MA, U.S.A.).

### Western blot analysis

After treatment, the cells were collected, washed and lysed with ice-cold RIPA lysis buffer (Beyotime Inst. Biotech) with 1 mmol/l PMSF. Protein concentrations were calculated using BCA assay kits (Beyotime Inst. Biotech). Total cellular protein (20 μg) was subjected to 12% SDS-PAGE and transferred to PVDF membranes (Millipore). The membranes were blocked with 5% nonfat milk powder at room temperature for 2 h, followed by immunoblotting with primary antibodies at 4°C overnight and immunoblotting with HRP-conjugated secondary antibody at room temperature for 1 h. Following each step, the membranes were washed three times with PBST for 5 min. Finally, the blots were developed using an enhanced chemiluminescence system (Pierce). Actin was used as a loading control.

### Colony formation assay

Five hundred exponentially growing cells were plated in six-well cell culture plates in a total volume of 2 ml of medium and incubated for 6 h. Next, 2 mM of KIF4A-siRNA was added to the medium, and incubation continued for 6 h. Following replacement with fresh medium, colony formation was monitored. Ten days later, the colonies were fixed with 75% ethanol and stained with methylene blue. The optical density (OD) values were detected using a microplate reader.

### Flow cytometry for quantitative analysis of apoptosis

After treatment, 3 × 10^5^ cells were collected used for each sample. For apoptosis detection, cells were stained using the Annexin V-FITC Apoptosis Detection Kit I (BD Biosciences, San Diego, CA) according to the manufacturer’s recommendation. The stained cells were determined by flow cytometry (BD FACS Canto) and analyzed by the FCS Express v2.0 software, as in our previous studies [28].

### Transwell assay

Cells were seeded in the chamber with 5 × 10^4^ cells in 100 μl FBS-free culture medium. A 600 μl volume of culture medium (10% FBS) was added to the 24-well plate. After a 24 h incubation, the suspended cells were washed and removed, then fixed with methylalcohol for 30 min. Subsequently, the non-migrated cells were removed and the migrated cells were stained with 1% Crystal Violet. The OD values were detected using a microplate reader as described in our previous studies [29].

### RNA interference to inhibit KIF4A expression

A mixture of 200 nM KIF4A-siRNA (KIF4A-siRNA-1: GGAACAGGGCAACAACTCT; KIF4A-siRNA-2: TAAGGATACCCTTCTATCT; KIF4A-siRNA-3:TGCTGTTTGAGGAACGAAA) (HonorGene, Changsha, China) and control siRNA were added to each well of a six-well plate for 6 h, after which fresh RPMI media containing 10% serum was added. Cells were harvested 48 h later.

### Statistical analysis

All statistical analyses were performed using SPSS 19.0 (SPSS Inc., Chicago, IL, U.S.A.). All of the data are presented as mean ± standard deviation (SD). Statistical significance comparing two groups was evaluated by the Student’s *t* test. A *P-*value<0.05 was considered statistically significant.

## Results

### Identification of DEGs from GSE85716, GSE32863, and GSE116959 datasets

**Figure 1:**
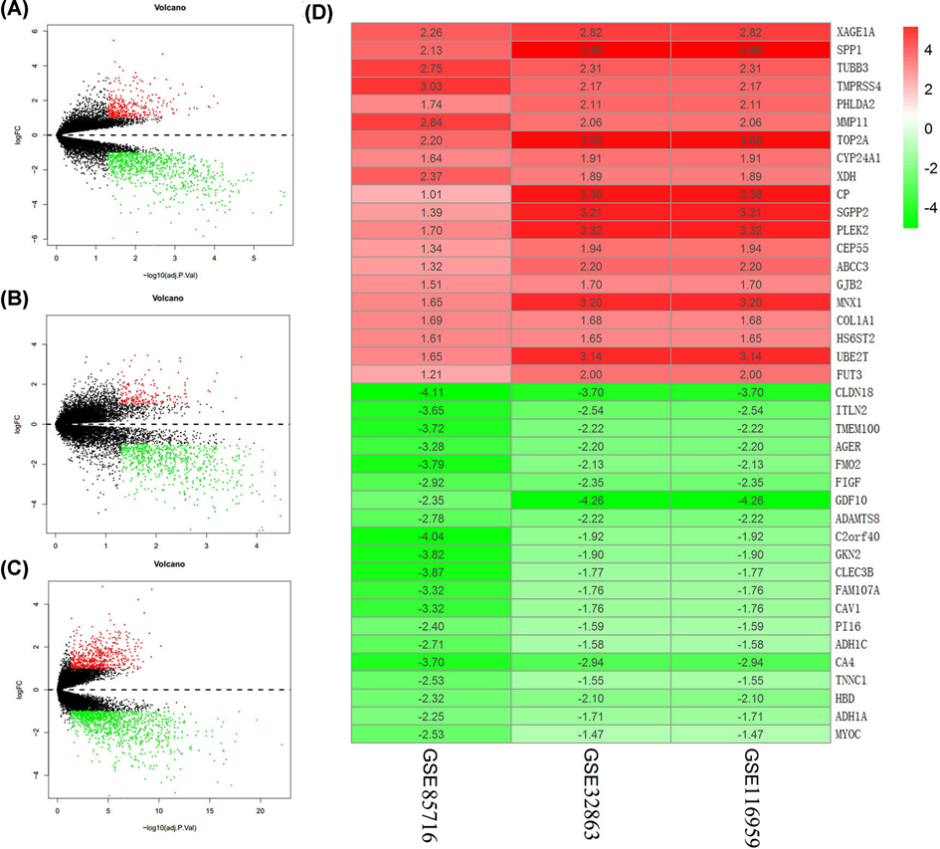
Identification of DEGs from GSE85716, GSE32863, and GSE116959 datasets (**A–C**) Volcano plot showing the DEGs of the GSE85716, GSE32863, and GSE116959 datasets. (**D**) Hierarchical clustering comparing the top 20 up-regulated and down-regulated genes in adjacent normal lung tissues and LUAD tissues.

To identify genes that might be involved in the tumorigenesis of LUAD, we analyzed the DEGs in the GSE85716, GSE32863, and GSE116959 datasets. We used a Volcano plot to present DEGs in LUAD and nontumor lung tissues based on the data from these three databases; the red dots indicated genes that were up-regulated in LUAD, and the green dots indicated genes that were down-regulated in LUAD (Figure 1A–C). Next, we screened the top 20 up-regulated and down-regulated genes in the GSE85716, GSE32863, and GSE116959 datasets, respectively, and the differential distribution of DEGs between adjacent normal lung and LUAD tissues was displayed using the hierarchical clustering (Figure 1D). The up- and downregulated DEGs are listed in Supplementary Tables S3 and 4.

### GO and KEGG analyses of robust DEGs in LUAD

**Figure 2:**
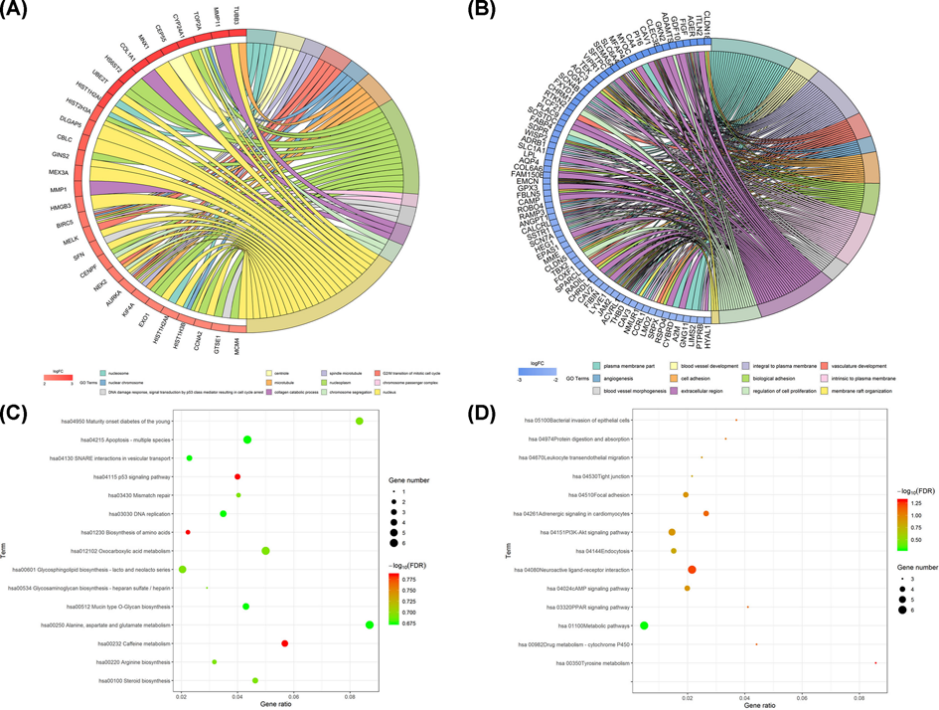
Functional characteristics analyses of robust DEGs GO enrichment analyses of (**A**) the up-regulated DEGs and (**B**) the down-regulated DEGs. KEGG pathway enrichment analyses of (**C**) the up-regulated DEGs and (**D**) the down-regulated DEGs.

Based on the expression profile of DEGs between adjacent normal lung and LUAD tissues, GO and KEGG analyses for DEGs were performed. The results of GO analysis revealed that the up-regulated DEGs were enriched in CC terms (nucleosome, centriole, spindle microtubule, nuclear chromosome, microtubule, nucleoplasm, chromosome passenger complex, and nucleus) and in BP terms (G2/M transition of mitotic cell cycle, DNA damage response, signal transduction by p53 class mediator resulting in cell cycle arrest, collagen catabolic process, and chromosome segregation) (Figure 2A). The down-regulated DEGs were enriched in CC terms (plasma membrane part, integral to plasma membrane, intrinsic to plasma membrane, and extracellular region) and BP terms (blood vessel development, vasculature development, angiogenesis, cell adhesion, biological, and blood vessel morphogenesis) (Figure 2B). The results of the GO function annotation for the up-regulated and down-regulated DEGs in LUAD are shown in Tables 1 and 2. Furthermore, KEGG pathway analysis disclosed that the up-regulated DEGs mainly participated in regulating glycosaminoglycan biosynthesis-heparan sulfate/heparin, mismatch repair, and biosynthesis of amino acids (Figure 2C); the down-regulated DEGs mainly participated in regulating neuroactive ligand–receptor interactions, metabolic pathways, and the PI3K-Akt signaling pathway (Figure 2D). The detailed KEGG pathways for the up-regulated and down-regulated DEGs in LUAD are shown in Tables 3 and 4.

**Table 1:** The GO analysis of the up-regulated DEGs in LUAD

Category	ID	Term	Genes	adj_pval
CC	GO:0000786	Nucleosome	HIST2H3A, HIST1H3B, HIST1H2AI, HIST1H2AM	0.0020621
CC	GO:0005814	Centriole	BIRC5, AURKA, CEP55, TOP2A	0.00347557
CC	GO:0005876	Spindle microtubule	KIF4A, BIRC5, AURKA	0.00623428
BP	GO:0000086	G2/M transition of mitotic cell cycle	NEK2, BIRC5, AURKA, MELK	0.00702927
CC	GO:0000228	Nuclear chromosome	HIST1H3B, BIRC5, TOP2A	0.00894277
CC	GO:0005874	Microtubule	KIF4A, NEK2, BIRC5, AURKA, TUBB3	0.00964457
CC	GO:0005654	Nucleoplasm	EXO1, HIST2H3A, GINS2, CYP24A1, KIF4A, CENPF, AURKA, BIRC5, MCM4, GTSE1, HIST1H3B, HS6ST2, CCNA2, TOP2A, UBE2T	0.01268473
CC	GO:0032133	Chromosome passenger complex	BIRC5, AURKA	0.01337317
BP	GO:0006977	DNA damage response, signal transduction by p53 class mediator resulting in cell cycle arrest	AURKA, SFN, GTSE1	0.01356678
BP	GO:0030574	Collagen catabolic process	COL1A1, MMP1, MMP11	0.01441139
BP	GO:0007059	Chromosome segregation	NEK2, CENPF, TOP2A	0.01616706
CC	GO:0005634	Nucleus	EXO1, HIST2H3A, CYP24A1, GINS2, HMGB3, NEK2, DLGAP5, MEX3A, CENPF, BIRC5, AURKA, SFN, MCM4, CBLC, MNX1, HIST1H2AI, HIST1H3B, HIST1H2AM, TOP2A, CCNA2, UBE2T, TUBB3, MELK	0.01726561

**Table 2:** The GO analysis of the down-regulated DEGs in LUAD

Category	ID	Term	Genes	adj_pval
CC	GO:0044459	Plasma membrane part	FXYD1, CAV3, CAV2, EMCN, CAV1, CLDN18, ACVRL1, LIMS2, CLDN5, SLC6A4, MME, AQP4, GNG11, VIPR1, NMUR1, SDPR, TEK, CALCRL, SCN7A, SLC1A1, CCRL1, PTPRB, RAMP3, AGER, LYVE1, THBD, ADRB1, SSTR1, CHRM1, CYBRD1, CA4, SCN4B, JAM2	0.00108239
BP	GO:0001568	Blood vessel development	SEMA5A, CAV1, EMCN, ACVRL1, LMO2, EPAS1, FOXF1, ROBO4, ANGPT1, FIGF	0.00230295
CC	GO:0005887	Integral to plasma membrane	FXYD1, PTPRB, RAMP3, CAV2, CAV1, ACVRL1, SLC6A4, AQP4, MME, VIPR1, AGER, LYVE1, ADRB1, THBD, SSTR1, NMUR1, CHRM1, TEK, SCN4B, SCN7A, CALCRL, JAM2, SLC1A1, CCRL1	0.00271098
BP	GO:0001944	Vasculature development	SEMA5A, TCF21, CAV1, EMCN, ACVRL1, LMO2, EPAS1, FOXF1, ROBO4, ANGPT1, FIGF	0.00271098
BP	GO:0001525	Angiogenesis	SEMA5A, EMCN, ACVRL1, EPAS1, ROBO4, ANGPT1, FIGF	0.00337538
BP	GO:0007155	Cell adhesion	CLDN18, EMCN, CLDN5, RADIL, SEMA5A, LYVE1, WISP2, SRPX, COL6A6, FBLN5, TEK, JAM2, MFAP4, AOC3	0.00362546
BP	GO:0022610	Biological adhesion	CLDN18, EMCN, CLDN5, RADIL, SEMA5A, LYVE1, WISP2, SRPX, COL6A6, FBLN5, TEK, JAM2, MFAP4, AOC3	0.00494697
CC	GO:0031226	Intrinsic to plasma membrane	FXYD1, PTPRB, RAMP3, CAV2, CAV1, ACVRL1, SLC6A4, AQP4, MME, VIPR1, AGER, LYVE1, ADRB1, THBD, SSTR1, NMUR1, CHRM1, TEK, SCN4B, SCN7A, CALCRL, JAM2, SLC1A1, CCRL1	0.00522618
BP	GO:0048514	Blood vessel morphogenesis	SEMA5A, CAV1, EMCN, ACVRL1, EPAS1, FOXF1, ROBO4, ANGPT1, FIGF	0.00561097
CC	GO:0005576	Extracellular region	A2M, EMCN, ITLN2, OGN, RSPO4, WISP2, COL6A6, SOSTDC1, GPX3, HEG1, SFTPC, ANGPT1, FIBIN, FAM150B, PI16, FIGF, MYOC, LPL, HYAL1, SPARCL1, CAMP, AGER, PLAC9, C2ORF40, GKN2, CHRDL1, ADAMTS8, THBD, CLEC3B, FBLN5, GDF10, MFAP4	0.00599981
BP	GO:0042127	Regulation of cell proliferation	HYAL1, CAV2, CAV1, ACVRL1, TBX2, VIPR1, AGER, WISP2, ADAMTS8, SSTR1, CHRM1, TEK, RTKN2, FABP4, CALCRL, FIGF	0.00656309
BP	GO:0031579	Membrane raft organization	CAV3, CAV2, CAV1	0.00679421

**Table 3:** The KEGG analysis of the up-regulated DEGs in LUAD

Term	Count	Ratio	FDR
Hsa00534 Glycosaminoglycan biosynthesis-heparan sulfate/heparin	1	0.02912621	0.20276459
Hsa03430 Mismatch repair	2	0.04032258	0.20276459
Hsa01230 Biosynthesis of amino acids	2	0.02234637	0.16376332
Hsa00100 Steroid biosynthesis	3	0.0462963	0.20276459
Hsa04115 p53 signaling pathway	3	0.04	0.16376332
Hsa04130 SNARE interactions in vesicular transport	3	0.02285714	0.21164483
Hsa00512 Mucin type O-Glycan biosynthesis	4	0.04301075	0.21164483
Hsa00601 Glycosphingolipid biosynthesis-lacto and neolacto series	5	0.02040816	0.20276459
Hsa04950 Maturity onset diabetes of the young	5	0.08333333	0.20276459
Hsa012102 Oxocarboxylic acid metabolism	6	0.05	0.20276459
Hsa00220 Arginine biosynthesis	2	0.03174603	0.20276459
Hsa04215 Apoptosis-multiple species	6	0.04347826	0.21164483
Hsa00250 Alanine, aspartate and glutamate metabolism	6	0.08695652	0.21164483
Hsa00232 Caffeine metabolism	4	0.05681818	0.16376332
Hsa03030 DNA replication	4	0.03488372	0.21164483

**Table 4:** The KEGG analysis of the down-regulated DEGs in LUAD

Term	Count	Ratio	FDR
Hsa04080 Neuroactive ligand-receptor interaction	6	0.02158273	0.06041271
Hsa01100 Metabolic pathways	6	0.00483871	0.50410934
Hsa04151 PI3K-Akt signaling pathway	5	0.01457726	0.12284827
Hsa04261 Adrenergic signaling in cardiomyocytes	4	0.02649007	0.07636768
Hsa04024 cAMP signaling pathway	4	0.0199005	0.12086446
Hsa04510 Focal adhesion	4	0.01941748	0.12086446
Hsa04144 Endocytosis	4	0.01515152	0.1506064
Hsa00350 Tyrosine metabolism	3	0.08571429	0.04917388
Hsa00982 Drug metabolism - cytochrome P450	3	0.04411765	0.07636768
Hsa03320 PPAR signaling pathway	3	0.04109589	0.07636768
Hsa05100 Bacterial invasion of epithelial cells	3	0.03703704	0.08027977
Hsa04974 Protein digestion and absorption	3	0.03333333	0.09115089
Hsa04670 Leukocyte transendothelial migration	3	0.025	0.12284827
Hsa04530 Tight junction	3	0.02158273	0.1506064

### Establishment of the PPI network and module analyses of genes in LUAD

**Figure 3:**
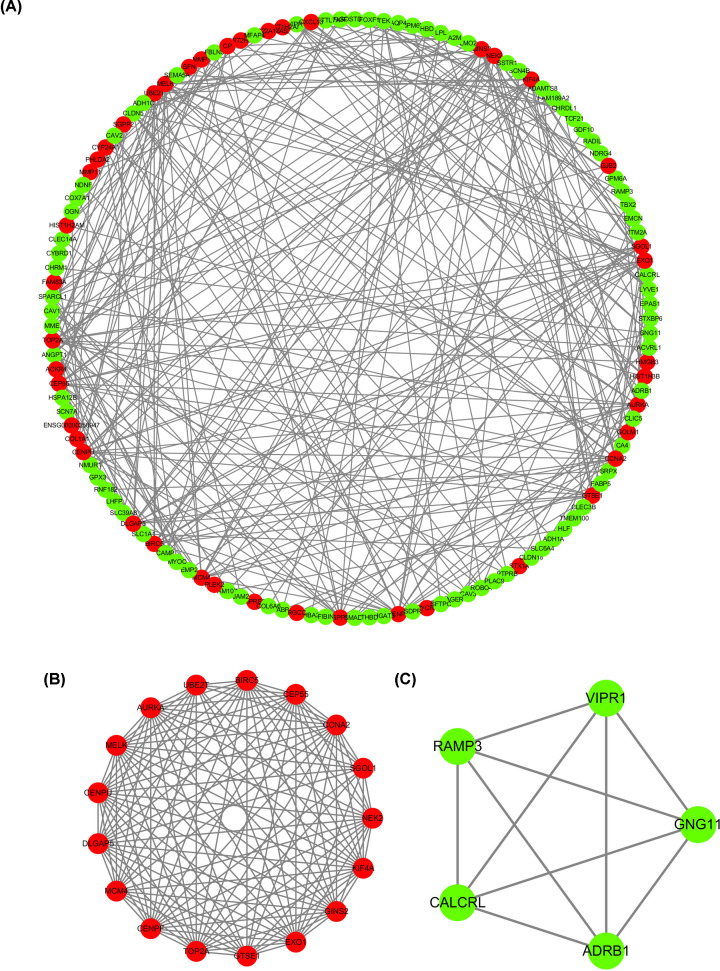
Establishment of the PPI network and modules analyses of genes in LUAD (**A**) The overall PPI network in LUAD. (**B**) The PPI network of module 1 in LUAD. (**C**) The PPI network of module 2 in LUAD.

To further predict the underlying functions of DEGs, we analyzed any potential co-expressed mRNAs of these DEGs and determined their associations by PPIs. As presented in Figure 3A, the entire PPI network revealed potential interrelationships between a number of genes, which might provide a foundation for the pathway research in LUAD. Moreover, we also analyzed two of the modules, and the relative networks are displayed in Figure 3B,C. Genes with the most highly connected cluster were extracted using the MCODE plug-in in Cytoscape after PPI networks were analyzed. The DMNC and MCC algorithms of Cytoscape were used to identify hub genes. The top ten hub genes based on the two methods were screened, and seven mutual hub genes were isolated, including BIRC5, DLGAP5, CENPF, KIF4A, TOP2A, AURKA, and CCNA2. These seven genes were all significantly up-regulated in LUAD samples compared with normal samples and were used for further analysis.

### Validation of seven hub genes expression in LUAD samples based on TCGA database

**Figure 4:**
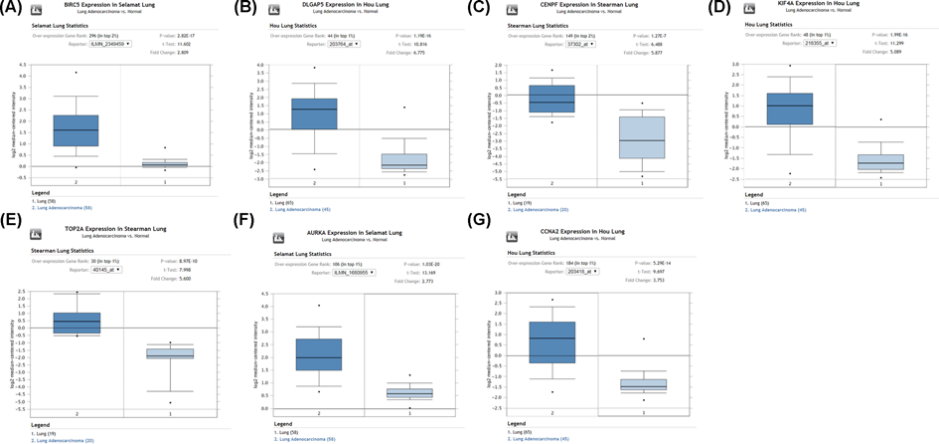
Validation of the expression of seven hub genes in LUAD samples based on the Oncomine database The expression levels of (**A**) BIRC5, (**B**) DLGAP5, (**C**) CENPF, (**D**) KIF4A, (**E**) TOP2A, (**F**) AURKA, and (**G**) CCNA2 in LUAD were obtained from the Oncomine database.

**Figure 5:**
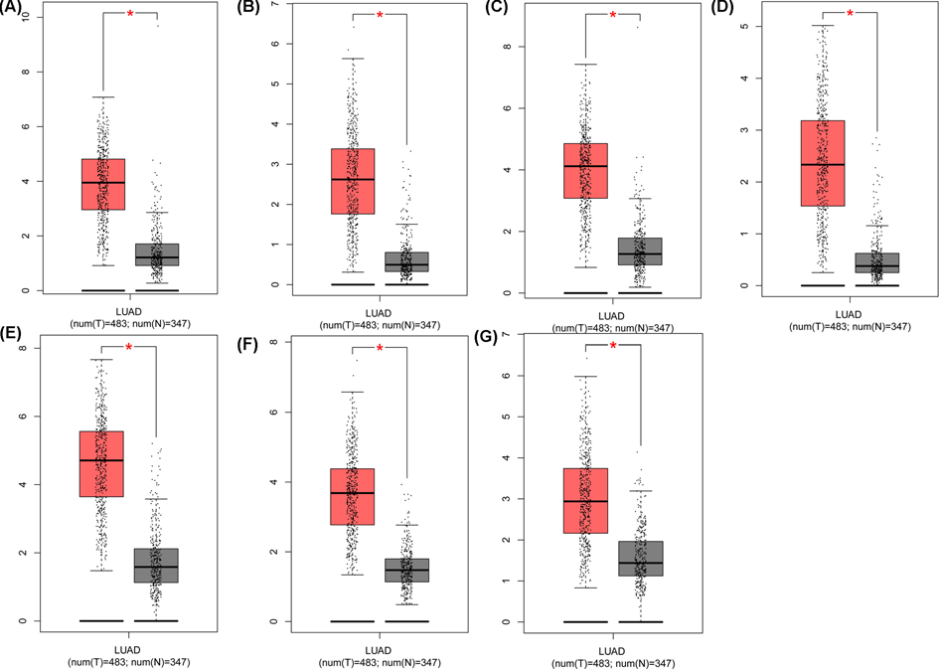
Identification of the expression of the seven hub genes in LUAD samples in the TCGA database Box plots show the expression of (**A**) BIRC5, (**B**) DLGAP5, (**C**) CENPF, (**D**) KIF4A, (**E**) TOP2A, (**F**) AURKA, and (**G**) CCNA2 in LUAD from the TCGA database. *P<0.05.

We utilized the Oncomine database to further reveal the expression of these seven hub genes in LUAD. The expression of BIRC5, DLGAP5, CENPF, KIF4A, TOP2A, AURKA, and CCNA2 (Figure 4) was significantly higher in LUAD tissue samples than that in nontumor lung tissue samples. Meanwhile, TCGA database was used to further identify the levels of the seven hub genes in LUAD. The results from the TCGA database displayed the same expression trends as in the Oncomine database (Figure 5). Therefore, these seven hub genes were significantly highly expressed in LUAD.

### Overall survival and disease-free survival analyses of seven hub genes in LUAD

**Figure 6:**
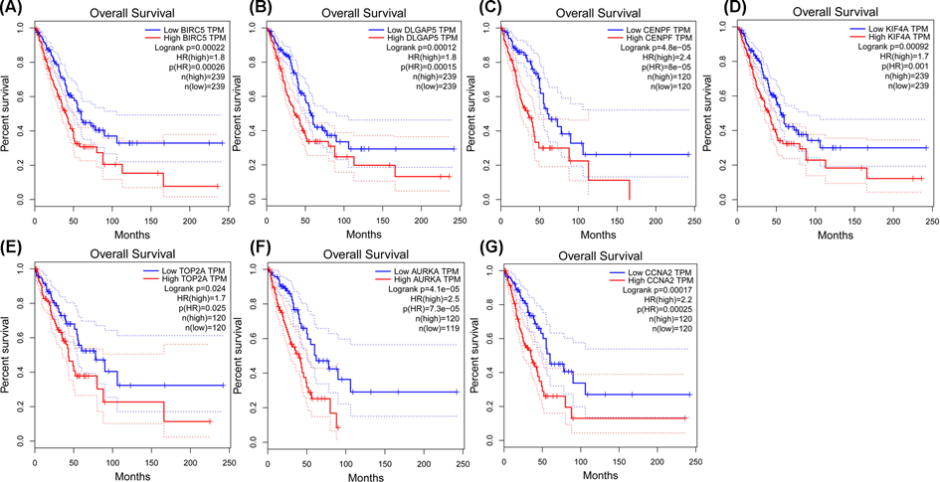
Overall survival analyses of the seven hub genes in LUAD on the basis of the TCGA and GTEx data in GEPIA Statistical graphs indicate the results of overall survival analyses of (**A**) BIRC5, (**B**) DLGAP5, (**C**) CENPF, (**D**) KIF4A, (**E**) TOP2A, (**F**) AURKA, and (**G**) CCNA2 in LUAD. Red lines represent the high expression of genes and the blue lines represent low expression of genes.

**Figure 7:**
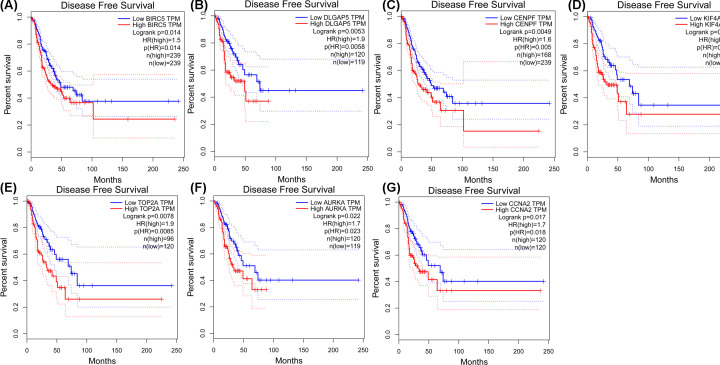
Disease-free survival analyses of the seven hub genes in LUAD based on TCGA and GTEx data in GEPIA In accordance with the TCGA and GTEx data in GEPIA, the disease-free survival of the seven hub genes are shown: (**A**) BIRC5, (**B**) DLGAP5, (**C**) CENPF, (**D**) KIF4A, (**E**) TOP2A, (**F**) AURKA, and (**G**) CCNA2.

To further assess the prognostic values of the seven hub genes in LUAD, TCGA, and GTEx datasets were obtained from GEPIA. The results of the overall survival analyses revealed that the high expression of BIRC5, DLGAP5, CENPF, KIF4A, TOP2A, AURKA, and CCNA2 (Figure 6) was associated with shorter overall survival than in patients with low expression. Simultaneously, the data also indicated that high expression of the seven hub genes could shorten the disease-free survival in LUAD (Figure 7). Therefore, the seven hub genes were associated with poor prognosis of LUAD.

### CENPF, DLGAP5, and KIF4A expression was positively correlated with the clinical stage of LUAD

**Figure 8:**
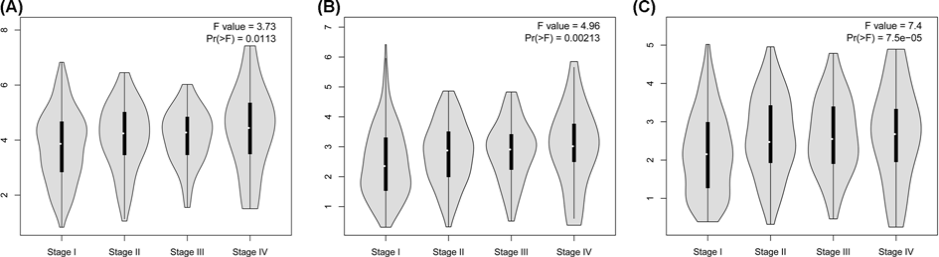
CENPF, DLGAP5, and KIF4A expression was positively correlated with the clinical stage of LUAD The levels of CENPF, DLGAP5, and KIF4A expression were analyzed in different stages of LUAD.

We screened the genes (CENPF, DLGAP5, and KIF4A), which were most likely to be associated with LUAD and analyzed any possible relationships with the clinical stage of LUAD. As displayed in Figure 8, the expression levels of CENPF, DLGAP5, and KIF4A were dramatically increased in the different stages of LUAD, and the expression of CENPF, DLGAP5, and KIF4A were gradually increased as the LUAD stages increased. Thus, we suggested that high expression of CENPF, DLGAP5, and KIF4A might be associated with higher stages of LUAD. Meanwhile, the seven hub genes including KIF4A, CENPF, and DLGAP5 contributed mostly to the GO analysis results (Figure 2A, Table 1).

### Expression validation of the seven hub genes by qRT-PCR

We compared the mRNA expression levels of the seven hub genes in LUAD tissues and paired normal tissues. As shown in Supplementary Figure S1, compared with their expression in paired normal samples, the expression of BIRC5, DLGAP5, CENPF, KIF4A, TOP2A, AURKA, and CCNA2 was significantly increased in LUAD samples (*P*<0.01).

### KIF4A was highly expressed in LUAD cells

**Figure 9:**
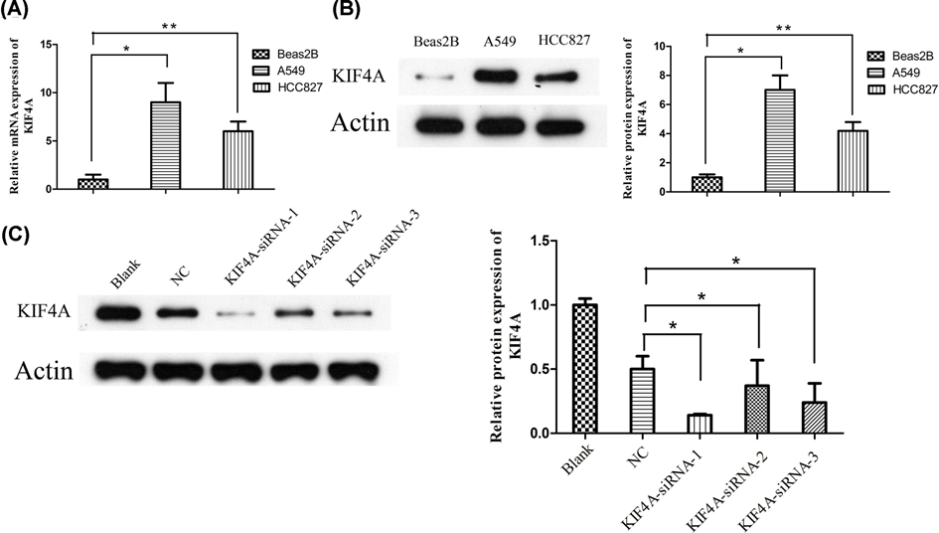
KIF4A was significantly up-regulated in LUAD cells (**A**) mRNA expression levels of KIF4A in the LUAD cells were higher than those in normal bronchial epithelial cells. (**B**) Expression of KIF4A protein in the LUAD cell lines was higher than in normal bronchial epithelial cells. (**C**) The protein expression levels of KIF4A targeted by siRNA-1, siRNA-2, and siRNA-3. *, *P*-value<0.05; **, *P-*value<0.01.

After identifying the seven hub genes and given the limited number of studies focusing on the relationship between KIF4A and the progression of LUAD, we further studied the expression and function of KIF4A *in vitro*. As shown in Figure 9A,B, the mRNA and protein expression levels of KIF4A were upregulated in LUAD cells (HCC827 and A549) compared with that in normal bronchial epithelial cells Beas2B.

### Knockdown of KIF4A decreased the migration in A549 cell lines

**Figure 10:**
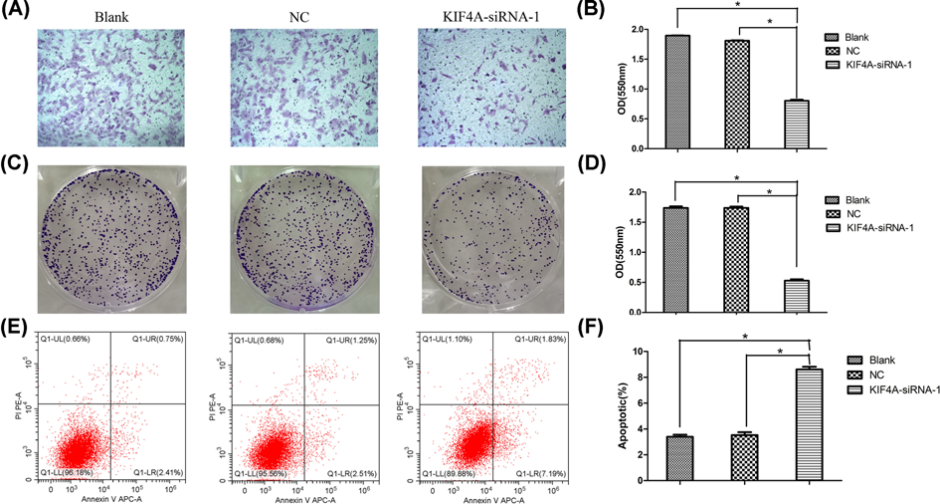
Silencing KIF4A inhibited proliferation, migration, and promoted apoptosis in the A549 cell line (**A** and **B**) Silencing KIF4A suppressed the migration of A549 cells. (**C** and **D**) KIF4A knockdown decreased cell proliferation capacity of A549 cells. (**E** and **F**) KIF4A knockdown promoted the apoptotic rate of A549 cells; *, *P-*value<0.05.

To further confirm the role of KIF4A, siRNA experiments were performed in A549 cells. The efficiency of the knockdown of KIF4A expression was examined by Western blotting, as shown in Figure 9C. To investigate the relationship between KIF4A and migration, we conducted the Transwell assay. The results revealed that the OD value of migrated KIF4A-siRNA-1 cells was decreased significantly, compared with that in the blank and the negative control groups (*P*<0.05) (Figure 10A,B).

### Effect of KIF4A on the proliferation and apoptosis of A549 cell lines

The colony formation assay showed that the OD values of the clone numbers decreased significantly in the KIF4A-siRNA-1 cells compared with that of the blank group and the negative control group (*P*<0.05) (Figure 10C,D). In addition, the apoptosis rate of the KIF4A-siRNA-1 cells increased significantly compared with that in control group as determined by the flow cytometry assay (*P*<0.05) (Figure 10E,F). Together, these results indicated that KIF4A suppression decreased cell proliferation and increased apoptosis abilities in A549 cell lines.

## Discussion

In recent years, with the development of high-throughput sequencing and bioinformatics, a novel research model was formed [30,31]. Massive quantities of research data generated by microarrays have been uploaded to the GEO database for sharing, and scientific researchers classified and downloaded the relevant data in accordance with their area of interest [32]. Meanwhile, researchers have further integrated and compared similar experimental data carried out by different laboratories, so as to unearth potentially useful data.

LUAD is a type of lung cancer with a high degree of malignancy, poor prognosis, and characterized by the increased likelihood of developing chest, liver, bone, and even intracranial metastasis [33,34]. At present, the majority of LUAD patients are diagnosed in the advanced stages and cannot be treated with surgery due to the presence of distant metastasis [35]. Although chemoradiotherapy can improve symptoms, the adverse reactions are significant [36]. Therefore, attempts at improving the survival rate of patients have become the main purpose of LUAD treatment. High-throughput genetic sequencing and bioinformatics might provide a new approach for the study of LUAD pathogenesis.

In our study, we selected ‘LUAD’ as the keyword to search for related datasets from the GEO datasets, and then performed a detailed biological analysis of these DEGs. Subsequently, in order to better understand the DEGs and their associated gene function, related pathways, protein interaction in LUAD, we conducted bioinformatics studies on LUAD using GO functional annotation, KEGG pathway analysis, and PPIs analysis. Overall, our results may provide a theoretical basis for exploring the pathogenesis of LUAD and further studies.

The results of the GO function annotation revealed that the upregulated DEGs in LUAD were mainly concentrated in the nucleosome, centriole, and spindle microtubule, while the down-regulated DEGs in LUAD were mainly enriched in the plasma membrane, in blood vessel development, and in functions integral to the plasma membrane. Meanwhile, KEGG pathway analysis revealed that the up-regulated DEGs in LUAD were mainly enriched in alanine, aspartate, and glutamate metabolism, and oxocarboxylic acid metabolism, while the downregulated DEGs in LUAD were mainly enriched in neuroactive ligand–receptor interactions and in tyrosine metabolism. Moreover, we obtained the key DEGs in LUAD using PPI network analysis.

With the intensive study of the PPI network, the topological structure of the PPI network obtained is frequently associated with disease-related proteins [37,38]. Previous studies have documented that the proteins in the same disease are more likely to interact and be co-expressed in the network [39], while the functional changes of neighboring node proteins associated with a disease are more likely to cause the same or similar diseases [40,41]. Meanwhile, the smaller nodes that interact with numerous proteins in the protein network are called hubs [42]. Hub genes may have more crucial biological functions in disease development. In biological networks, the change in expression of hub proteins will generate serious destruction of the connected network and may seriously affect the biological functions of cells [43]. Currently, a large number of key hub genes have been identified in a variety of cancers, such as hepatocellular carcinoma [44], bladder cancer [45], renal cell carcinoma [46], and osteosarcoma [47]. However, a number of hub genes in LUAD remain undetermined. In our study, we have filtered seven key hub genes including BIRC5, DLGAP5, CENPF, KIF4A, TOP2A, AURKA, and CCNA2 in LUAD. Simultaneously, we found that these seven hub genes were significantly up-regulated in LUAD using Oncomine data and TCGA datasets, and up-regulation of these seven hub genes were associated with a poor prognosis of LUAD. In addition, we determined that the expression of CENFP, KIF4A, and DLGAP5 might be associated with the metastasis of LUAD. Moreover, we further confirmed the increased mRNA expression level of these seven key hub genes in primary LUAD samples compared with paired normal samples.

In the present study, we provided evidence that KIF4A was significantly up-regulated in LUAD cells. To date, only one study has reported that the knockdown KIF4A might suppress the growth of NSCLC cell lines [48]. As for other tumor types, KIF4A has been reported to enhance carcinoma development by promoting cell cycle progression *in vitro* and *in vivo* in colorectal cancer and hepatocellular carcinoma [49,50]. Our study revealed that silencing KIF4A expression inhibited proliferation, migration, and promoted apoptosis in A549 cell lines, which was in agreement with the previous studies indicated above.

## Conclusions

We have identified new DEGs and pathways active in LUAD, and more specifically, KIF4A as a hub gene with the capacity to promote the progression of LUAD. Our findings provide researchers with a potential therapeutic target for molecular cancer therapy.

## Supplementary Material

Supplementary Figure S1 and Tables S1-S4

## Data Availability

The datasets used or analyzed during the current study are available from the GEO (https://www.ncbi.nlm.nih.gov/geo/), Oncomine (https://www.oncomine.org/resource/login.html), GEPIA (http://gepia.cancer-pku.cn/), STRING (https://string-db.org/), KOBAS (http://kobas.cbi.pku.edu.cn/), DAVID (https://david.ncifcrf.gov/), and TCGA (https://cancergenome.nih.gov/) databases.

## Abbreviations

DEG: differentially expressed gene
GEO: gene expression omnibus
LUAD: lung adenocarcinoma
PPI: protein–protein interaction
qRT-PCR: real-time quantitative PCR
STRING: Search Tool for the Retrieval of Interacting Genes
