# Supplementary material for: Identification of KIF4A and its effect on the progression of lung adenocarcinoma based on the bioinformatics analysis
Source: Biosci Rep. 2021 Jan 22;41(1):BSR20203973. doi: 10.1042/BSR20203973 (PMC7823194; doi:10.1042/BSR20203973)
Supplement: Supplementary Figure S1 and Tables S1-S4 [file BSR-2020-3973_supp.pdf]

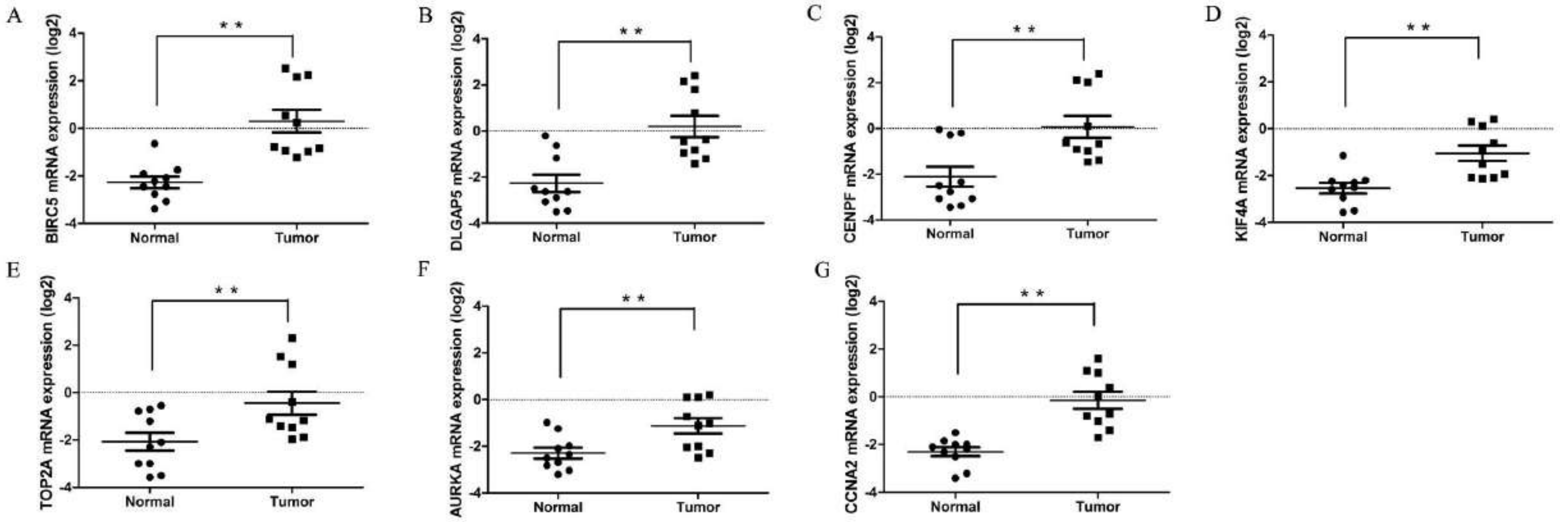
A
BIRC5 mRNA expression (log2)
4
2
0
-2
-4
Normal
Tumor
* *
B
DLGAP5 mRNA expression (log2)
Normal
Tumor
* *
C
CENPF mRNA expression (log2)
Normal
Tumor
* *
D
KIF4A mRNA expression (log2)
Normal
Tumor
* *
E
TOP2A mRNA expression (log2)
Normal
Tumor
* *
F
AURKA mRNA expression (log2)
Normal
Tumor
* *
G
CCNA2 mRNA expression (log2)
Normal
Tumor
* *

Supplementary Figure 1. Validation of the mRNA expression of the seven hub genes comparing paired normal samples (n=10) and LUAD samples (n=10) by PCR analysis. (A) BIRC5, (B) DLGAP5, (C) CENPF, (D) KIF4A, (E) TOP2A, (F) AURKA, and (G) CCNA2. All of the data are presented as means ± SD. **, *P-value*<0.01.

Supplementary Table 1. The detailed information of the three GEO datasets.

Dataset	Numbers of samples (Tumor / Normal)	Histological types	Experiment type	Origin
GSE85716	12/12	Adenocarcinoma	mRNA	Sci Rep
GSE32863	58/58	Adenocarcinoma	mRNA	Genome Res
GSE116959	57/11	Adenocarcinoma	mRNA	Oncogene

Note: GEO, Gene Expression Omnibus

Supplementary Table 2. The primer sequences of the hub genes.

BIRC5	forward, 5′-ATTCGTCCGGTTGCGCTTTCC-3′
	reverse, 5′-CACGGCGCACTTTCTTCGCAG-3′
DLGAP5	forward, 5′-AAGTGGGTCGTTATAGACCTGA-3′
	reverse 5′-TGCTCGAACATCACTCTCGTTAT-3′
CENPF	forward, 5′-AAAGAAACAGACGGAACAACTG-3′
	reverse 5′-CCAAGCAAAGACCGAGAACT-3′
KIF4A	forward, 5′-TACTGCGGTGGAGCAAGAAG-3′
	reverse 5′-CATCTGCGCTTGACGGAGAG3′
TOP2A	forward, 5′-CTAGTTAATGCTGCGGACAACA-3′
	reverse 5′-CATTTCGACCACCTGTCACTT-3′
AURKA	forward, 5′-AGTTGGAGGTCCAAAACGTG-3′
	reverse 5′-TCCAAGTGGTGCATATTCCA-3′
CCNA2	forward, 5′-CACTCTACACAGTCAGGGGA-3′
	reverse 5′-AGTGTCTCTGGTGGGTTGAG-3′
GADPH	forward 5′-CATCTCTGCCCCCTCTGCTGA-3′
	reverse 5′-GGATGACCTTGCCCACAGCCT-3′

Supplementary Table 3. The information of the upregulated DEGs between adjacent normal lung and LUAD tissues

Gene	logFC	AveExpr	t	P.Value	adj.P.Val	B
XAGE1A	2.818124	7.024731	3.59438	0.003301	0.035687	-2.03285
SPP1	3.67501	8.270667	6.117881	3.79E-05	0.002047	2.440225
TUBB3	2.527322	4.812491	3.673739	0.002838	0.032767	-1.88325
TMPRSS4	2.936224	7.172396	3.906683	0.001826	0.02494	-1.44526
PHLDA2	2.070969	6.68861	4.797153	0.000355	0.009232	0.191714
MMP11	2.36827	8.729277	13.11481	2.51E-07	0.000204	7.533217
TOP2A	3.364929	5.70608	4.614423	0.000494	0.01121	-0.13769
CYP24A1	1.814183	3.943949	3.853667	0.002018	0.026237	-1.54473
XDH	2.270497	4.378626	3.70179	0.002691	0.031642	-1.8304
CP	3.270281	6.584131	3.323473	0.005542	0.048053	-2.54374
SGPP2	3.104349	6.703745	6.3222	2.74E-05	0.001634	2.765672
PLEK2	2.177398	4.160249	4.978233	0.000258	0.007444	0.514129
CEP55	1.549506	4.897171	3.863248	0.001982	0.026053	-1.52675
ABCC3	2.004989	8.640878	5.08149	0.000215	0.006641	0.696099
GJB2	1.599979	3.840176	3.643585	0.003006	0.034034	-1.94008
MNX1	2.994084	3.579718	3.925726	0.001762	0.024347	-1.40956
COL1A1	1.690818	7.455179	3.372773	0.005042	0.045648	-2.45084
HS6ST2	1.688701	4.689706	4.838842	0.00033	0.008818	0.266306
UBE2T	2.967068	5.103812	5.459279	0.000112	0.004273	1.349591
FUT3	2.043435	5.736336	5.475034	0.000109	0.004223	1.376411
STX1A	2.935411	5.067663	7.254678	6.67E-06	0.000628	4.173654
HIST1H2AI	2.880545	5.306701	4.208568	0.001038	0.01774	-0.88221
CXCL13	2.870715	4.146376	4.44511	0.000672	0.013532	-0.44627
GPT2	2.783919	4.724283	8.466538	1.26E-06	0.000211	5.822701
FAM83A	2.707739	5.976604	6.542513	1.94E-05	0.001292	3.109719
LOC100130811	2.680684	7.72016	4.785064	0.000363	0.009328	0.170044
HIST2H3A	2.61482	8.421248	3.836221	0.002086	0.02676	-1.5775
DLGAP5	2.58805	3.474919	4.088373	0.001299	0.02047	-1.10561
CBLC	2.585204	3.886146	4.999561	0.000248	0.007272	0.55183
GINS2	2.533287	5.133182	3.601494	0.003256	0.035532	-2.01944
MEX3A	2.527057	3.452469	4.490277	0.000618	0.012874	-0.36365
MMP1	2.510838	5.588877	3.448129	0.004365	0.041578	-2.30874
PYCR1	2.406433	6.333929	7.417624	5.28E-06	0.000547	4.406941
STK32A	2.333879	6.39677	5.765152	6.72E-05	0.003005	1.863954
HMGB3	2.330866	6.033155	5.581962	9.12E-05	0.003715	1.557512
GOLM1	2.317666	8.311508	7.010564	9.55E-06	0.000807	3.817162
BIRC5	2.294236	5.50253	3.487856	0.004045	0.039984	-2.2338
MELK	2.269227	4.768067	3.718442	0.002607	0.030877	-1.79904
SFN	2.266313	9.028779	5.394662	0.000125	0.00461	1.239219
CENPF	2.264698	5.174452	3.969908	0.001621	0.023304	-1.32683
PODXL2	2.259731	5.790414	9.528018	3.33E-07	0.000104	7.114909

NEK2	2.215783	3.851914	3.383537	0.004939	0.044964	-2.43055
AURKA	2.178471	6.812019	5.083053	0.000215	0.006635	0.698841
KIF4A	2.160272	5.144072	3.417562	0.004627	0.04325	-2.36639
LOC283710	2.151973	3.818108	5.34134	0.000137	0.004939	1.1477
EXO1	2.125513	4.785475	3.954176	0.00167	0.023572	-1.35628
HIST1H2AM	2.091739	6.882594	3.705284	0.002673	0.031522	-1.82382
MLF1IP	2.088122	4.340229	4.529615	0.000576	0.012375	-0.29187
GALNT7	2.084426	5.156065	6.720718	1.48E-05	0.001086	3.382813
HIST1H3B	2.082695	10.38385	3.40007	0.004785	0.044222	-2.39938
CCNA2	2.058195	6.612935	3.869014	0.00196	0.0259	-1.51592
GTSE1	2.027084	4.658961	3.294916	0.005853	0.049899	-2.59752
IL2RA	2.02583	5.918288	4.039577	0.001423	0.021402	-1.19661
SGOL1	2.021829	3.800428	3.638276	0.003036	0.034211	-1.95009
SLC22A18AS	2.00686	4.493097	3.537846	0.003677	0.038006	-2.13949
MCM4	2.001465	7.018757	5.442296	0.000116	0.004328	1.32064

Supplementary Table 4. The information of the downregulated DEGs between adjacent normal lung and LUAD tissues

Gene	logFC	AveExpr	t	P.Value	adj.P.Val	B
CLDN18	-3.88033	10.80772	-6.77385	3.46E-09	2.33E-07	10.76398
ITLN2	-3.51928	8.32816	-10.0317	4.25E-15	2.62E-12	24.02374
TMEM100	-3.35949	7.811716	-13.8606	1.37E-21	7.33E-18	38.49646
AGER	-3.09523	9.528863	-9.46257	4.47E-14	1.65E-11	21.73303
FMO2	-2.80928	9.846211	-10.3868	9.93E-16	7.41E-13	25.43872
FIGF	-2.70543	8.339863	-8.58609	1.74E-12	3.60E-10	18.1637
GDF10	-3.69388	8.413394	-9.57906	2.76E-14	1.16E-11	22.20396
ADAMTS8	-2.57774	8.193042	-8.38423	4.06E-12	7.24E-10	17.33719
C2orf40	-3.32916	8.144675	-7.6599	8.53E-11	9.85E-09	14.36906
GKN2	-2.58148	8.152254	-6.03009	7.30E-08	3.10E-06	7.80171
CLEC3B	-2.56803	12.07937	-8.41236	3.61E-12	6.58E-10	17.45243
FAM107A	-2.55681	8.368239	-8.62986	1.45E-12	3.02E-10	18.34278
CAV1	-2.55138	10.62217	-9.49008	3.99E-14	1.50E-11	21.84431
PI16	-2.23703	7.731624	-8.79103	7.37E-13	1.67E-10	19.00156
ADH1C	-2.46707	10.04903	-6.13795	4.71E-08	2.16E-06	8.225839
CA4	-3.45929	7.226869	-13.2789	1.18E-20	4.25E-17	36.42023
TNNC1	-2.41189	9.342043	-7.93431	2.69E-11	3.71E-09	15.49313
HBD	-2.38861	12.49211	-6.20956	3.52E-08	1.69E-06	8.508619
ADH1A	-2.03745	8.210561	-7.64582	9.05E-11	1.03E-08	14.31145
MYOC	-2.32466	6.28135	-12.5705	1.74E-19	3.48E-16	33.82893
C10orf116	-3.27965	12.82333	-7.48733	1.76E-10	1.76E-08	13.66327
MFAP4	-3.26452	12.49525	-7.38161	2.74E-10	2.54E-08	13.23151
SLC6A4	-3.25955	6.467535	-13.5003	5.19E-21	2.38E-17	37.21581
HBA2	-3.15205	12.25895	-6.23993	3.11E-08	1.51E-06	8.628815
MGAT3	-3.12558	8.290119	-8.5317	2.19E-12	4.30E-10	17.94111
SEMA5A	-3.10479	9.905222	-8.71726	1.00E-12	2.12E-10	18.70017
SFTPC	-3.09917	15.7502	-3.37863	0.001203	0.009561	-1.48333
VIPR1	-2.99041	7.810181	-10.4062	9.18E-16	7.01E-13	25.51536
DNASE1L3	-2.98458	7.885619	-8.05608	1.61E-11	2.36E-09	15.99227
TEK	-2.91956	9.15555	-8.39065	3.96E-12	7.13E-10	17.36351
AOC3	-2.91684	10.50087	-8.86689	5.36E-13	1.27E-10	19.31132
SLC39A8	-2.91019	9.620007	-9.96957	5.49E-15	3.00E-12	23.77495
OGN	-2.89869	6.90445	-9.2366	1.14E-13	3.50E-11	20.81681
SCN4B	-2.8927	7.849193	-9.74378	1.39E-14	6.77E-12	22.86814
FXYD1	-2.89169	7.51509	-10.3037	1.39E-15	9.73E-13	25.10874
CHRM1	-2.8862	6.025893	-13.2775	1.19E-20	4.25E-17	36.4151
RTKN2	-2.8333	7.255558	-13.2013	1.58E-20	4.62E-17	36.13963
EMP2	-2.80704	12.36905	-10.1903	2.22E-15	1.45E-12	24.65726
TCF21	-2.79398	7.099916	-11.1203	5.12E-17	6.32E-14	28.3203
LAMP3	-2.76558	12.05272	-7.11124	8.49E-10	6.92E-08	12.13018
PLAC9	-2.7242	7.955677	-10.9874	8.73E-17	9.34E-14	27.8024

TPPP3	-2.70132	9.710932	-5.78585	1.95E-07	7.15E-06	6.84971
SOSTDC1	-2.67723	6.454697	-7.07564	9.86E-10	7.86E-08	11.98555
C13orf15	-2.67539	11.40839	-9.12861	1.80E-13	5.14E-11	20.37784
HLF	-2.66677	8.165715	-6.61809	6.59E-09	4.04E-07	10.13715
FABP4	-2.65189	6.410904	-11.0866	5.86E-17	6.96E-14	28.18919
SDPR	-2.64536	8.993928	-8.86357	5.44E-13	1.27E-10	19.29776
CDO1	-2.63628	7.183343	-9.46852	4.36E-14	1.63E-11	21.75711
WISP2	-2.62175	10.39802	-6.43426	1.40E-08	7.70E-07	9.401333
ADRB1	-2.62134	8.288305	-6.26603	2.80E-08	1.38E-06	8.732224
SLC1A1	-2.61163	8.777875	-7.06013	1.05E-09	8.29E-08	11.92259
SMAD6	-2.58313	8.978113	-9.53131	3.36E-14	1.31E-11	22.01104
GPM6A	-2.57284	6.375643	-17.7094	2.54E-27	8.15E-23	51.06694
KIAA0408	-2.56803	7.472079	-8.92201	4.26E-13	1.06E-10	19.5362
ANXA3	-2.56381	9.819399	-7.75614	5.70E-11	6.87E-09	14.76309
LPL	-2.53862	9.809238	-5.69038	2.85E-07	9.85E-06	6.481064
AQP4	-2.51776	8.945153	-6.6101	6.81E-09	4.15E-07	10.1051
COL6A6	-2.50901	7.426245	-6.9749	1.50E-09	1.13E-07	11.57684
HIGD1B	-2.50421	8.715539	-7.84493	3.92E-11	5.07E-09	15.12682
FAM150B	-2.45985	6.972387	-9.54918	3.12E-14	1.24E-11	22.08324
EMCN	-2.44193	8.931621	-7.43292	2.21E-10	2.14E-08	13.44098
NDRG4	-2.43241	8.797698	-8.15326	1.07E-11	1.69E-09	16.39064
FLJ46446	-2.42772	7.66975	-7.76319	5.53E-11	6.74E-09	14.79199
GPX3	-2.40849	9.617297	-6.54139	9.04E-09	5.26E-07	9.829621
CLIC5	-2.39573	10.07205	-7.91113	2.97E-11	4.04E-09	15.39811
FBLN5	-2.38828	10.36003	-7.7953	4.83E-11	5.98E-09	14.92349
ARHGEF26	-2.37306	8.088031	-9.21076	1.27E-13	3.86E-11	20.71186
CAMP	-2.36179	7.088716	-6.85472	2.47E-09	1.72E-07	11.09048
ROBO4	-2.33942	8.99776	-8.75217	8.67E-13	1.89E-10	18.84284
RAMP3	-2.33174	7.34154	-9.42879	5.14E-14	1.81E-11	21.59626
MS4A7	-2.29388	10.658	-7.24186	4.92E-10	4.24E-08	12.66165
ANGPT1	-2.2899	8.331996	-8.08597	1.42E-11	2.12E-09	16.11476
CALCRL	-2.27672	7.736635	-9.5818	2.73E-14	1.16E-11	22.21502
SSTR1	-2.27103	7.010182	-7.12858	7.90E-10	6.50E-08	12.20069
SCN7A	-2.26995	7.324706	-8.85966	5.53E-13	1.28E-10	19.28179
HEG1	-2.2667	10.78066	-8.45665	3.00E-12	5.64E-10	17.63382
EPAS1	-2.26343	13.84718	-7.5337	1.45E-10	1.50E-08	13.85279
MME	-2.26028	7.445706	-7.45879	1.98E-10	1.96E-08	13.54665
CLDN5	-2.255	10.97108	-5.85337	1.49E-07	5.65E-06	7.111636
PTPN21	-2.25005	8.382917	-9.96854	5.52E-15	3.00E-12	23.77082
TBX2	-2.24897	11.12536	-7.1021	8.83E-10	7.15E-08	12.09306
STXBP6	-2.24889	7.517232	-7.50572	1.63E-10	1.67E-08	13.7384
FOXF1	-2.24084	7.643346	-10.495	6.39E-16	5.19E-13	25.86736
SPARCL1	-2.2395	11.66687	-7.16908	6.67E-10	5.57E-08	12.3654
RADIL	-2.23148	7.865908	-5.35376	1.07E-06	3.02E-05	5.199254

HSPA12B	-2.22823	7.958093	-9.40991	5.56E-14	1.90E-11	21.51983
CHRDL1	-2.2239	8.296611	-7.29723	3.90E-10	3.49E-08	12.8873
CAV2	-2.21054	7.973899	-8.14635	1.10E-11	1.73E-09	16.36231
METTL7A	-2.20995	11.73772	-7.26409	4.49E-10	3.90E-08	12.75223
FIBIN	-2.20523	6.534673	-9.56318	2.94E-14	1.20E-11	22.13983
LYVE1	-2.16628	7.032022	-10.004	4.77E-15	2.78E-12	23.91293
JAM2	-2.16477	7.706179	-8.94995	3.79E-13	9.80E-11	19.65015
BTNL9	-2.16057	6.924479	-12.0282	1.41E-18	2.23E-15	31.80034
COX7A1	-2.15655	11.56955	-8.25141	7.10E-12	1.18E-09	16.79292
ACVRL1	-2.15443	10.51495	-7.40249	2.51E-10	2.35E-08	13.31672
THBD	-2.15159	11.43407	-6.61695	6.62E-09	4.05E-07	10.13258
CAV3	-2.1331	6.517061	-11.3989	1.69E-17	2.25E-14	29.39883
NMUR1	-2.13196	6.880116	-10.8567	1.48E-16	1.53E-13	27.29144
CLEC14A	-2.13112	7.950611	-8.17879	9.64E-12	1.52E-09	16.49527
GPM6B	-2.12317	7.24714	-9.29962	8.80E-14	2.82E-11	21.07268
RNF182	-2.11516	6.528325	-8.24481	7.30E-12	1.19E-09	16.76591
LHFP	-2.1079	11.25905	-8.01901	1.89E-11	2.69E-09	15.84028
CCRL1	-2.10416	7.283198	-8.53999	2.11E-12	4.18E-10	17.97503
FABP5	-2.09151	10.64035	-6.38179	1.74E-08	9.19E-07	9.192198
FAM189A2	-2.08542	8.003897	-7.54791	1.37E-10	1.43E-08	13.91091
NDNF	-2.06641	8.50587	-5.7008	2.74E-07	9.53E-06	6.52123
LMO2	-2.06471	10.74688	-6.84159	2.61E-09	1.81E-07	11.03739
SRPX	-2.04649	7.972472	-6.35074	1.98E-08	1.02E-06	9.068613
RSPO4	-2.04349	7.008409	-6.32377	2.21E-08	1.12E-06	8.961391
ITM2A	-2.04233	9.693858	-6.91608	1.92E-09	1.38E-07	11.33864
CYBRD1	-2.03853	9.845482	-6.85508	2.47E-09	1.72E-07	11.0919
A2M	-2.03499	14.49045	-5.88357	1.32E-07	5.15E-06	7.229095
GNG11	-2.03368	11.43239	-5.78635	1.94E-07	7.15E-06	6.851624
LIMS2	-2.01763	9.253783	-9.40687	5.63E-14	1.90E-11	21.50751
CCDC85A	-2.01239	6.916163	-8.90672	4.54E-13	1.11E-10	19.47384
PTPRB	-2.00856	7.921767	-8.25679	6.94E-12	1.16E-09	16.81501
HYAL1	-2.00272	8.545659	-5.64296	3.44E-07	1.16E-05	6.298781
